# Quantitative but not qualitative flavor recognition impairments in COVID-19 patients

**DOI:** 10.1007/s11845-021-02786-x

**Published:** 2021-09-25

**Authors:** Immacolata Cristina Nettore, Elena Cantone, Giuseppe Palatucci, Fabiana Franchini, Rufina Maturi, Mariagiovanna Nerilli, Elio Manzillo, Maria Foggia, Luigi Maione, Paola Ungaro, Annamaria Colao, Paolo Emidio Macchia

**Affiliations:** 1 Dipartimento di Medicina Clinica e Chirurgia, Università degli Studi di Napoli Federico II, Napoli, Italy; 2 Dipartimento di Neuroscienze e Scienze della Riproduzione e Odontostomatologiche, Università degli Studi di Napoli Federico II, Napoli, Italy; 3 Ospedale dei Colli, Napoli, Italy; 4 Istituto per l’Endocrinologia ed Oncologia Sperimentale (IEOS) G. Salvatore, CNR, Nazionale per le Ricerche, Napoli, Consiglio Italy

**Keywords:** COVID-19, Flavor, Flavor test, Hyposmia, Retro-nasal olfaction, Smell

## Abstract

**Background:**

Smell and taste dysfunctions (STDs) are symptoms associated with COVID-19 syndrome, even if their incidence is still uncertain and variable.

**Aims:**

In this study, the effects of SARS-CoV-2 infection on chemosensory function have been investigated using both a self-reporting questionnaire on smell and flavor perception, and a simplified flavor test.

**Methods:**

A total of 111 subjects (19 hospitalized [HOS] and 37 home-isolated [HI] COVID-19 patients, and 55 healthy controls [CTRL]) were enrolled in the study. They received a self-evaluation questionnaire and a self-administered flavor test kit. The flavor test used consists in the self-administration of four solutions with a pure olfactory stimulus (coffee), a mixed olfactory-trigeminal stimulus (peppermint), and a complex chemical mixture (banana).

**Results:**

After SARS-CoV-2 infection, HOS and HI patients reported similar prevalence of STDs, with a significant reduction of both smell and flavor self-estimated perception. The aromas of the flavor test were recognized by HI and HOS COVID-19 patients similarly to CTRL; however, the intensity of the perceived aromas was significantly lower in patients compared to controls.

**Conclusion:**

Data reported here suggests that a chemosensory impairment is present after SARS-CoV-2 infection, and the modified “flavor test” could be a novel self-administering objective screening test to assess STDs in COVID-19 patients.

Clinical trial registration no. NCT04840966; April 12, 2021, retrospectively registered

**Supplementary Information:**

The online version contains supplementary material available at 10.1007/s11845-021-02786-x.

## Introduction

In December 2019 a novel coronavirus, SARS-CoV-2, was first reported in China. The rapid spread of the virus and its substantial morbidity and mortality has prompted the World Health Organization to declare the coronavirus disease (COVID-19) outbreak a global pandemic [1].

Clinical manifestations of the infection range from mild to critical, even if the most serious effect of COVID-19 infection is interstitial pneumonia [2]. It is well known that viruses responsible for the common cold can cause post-viral smell impairment. Post-viral hypo/anosmia is one of the leading causes of smell deficiencies in adults [3]. However, while fever and cough are common symptoms of several viral infections, recent research indicated that sudden smell and taste dysfunctions (STDs) are cardinal, early, and potentially specific symptoms of COVID-19, also in otherwise asymptomatic individuals [4–6]. For instance, many scientific societies have published guidelines including STDs among the diagnostic criteria of COVID-19 [7, 8]. A possible reason for the olfactory impairment in COVID-19 patients is the high expression in nasal epithelial and olfactory cells of ACE2, the target protein used by SARS-CoV-2 to infect the host cells [9–12]. Olfactory dysfunction can be either conductive, mainly due to the physical blockage of odors in reaching the olfactory neuroepithelium, or sensorineural, due to the interruption of the route from olfactory receptors to the brain cortex, mainly caused by viral infections, head injuries, or neurodegenerative diseases. Previous studies proposed a propagation mechanism of SARS-CoV2, as well as other SARS-CoV infections, across the cribriform plate of the ethmoid bone, i.e., from the nose to the olfactory neuroepithelium, where ACE2 receptors are highly expressed. This viral neurotropism through the olfactory bulb is thought to be especially responsible for the post-COVID anosmia [12, 13].

The real incidence of STDs in COVID-19 patients is still uncertain and variable. Most of the published studies evaluated the presence of STDs using only questionnaire or phone/mail interviews, whereas only a few studies have used a direct evaluation with validated methods [14–21]. The use of specific chemosensory tests to evaluate STDs, although desirable, can imply several limitations. These include unnecessary time of physician’s exposure to virus, discomfort for patients (often in compromised general conditions), and high costs, also considering that the tests must be single-patient and non-reusable [14].

To better understand the COVID-related STDs, it is crucial to point out that most patients report a reduced/discontinued or distorted ability to taste flavors [22]. Flavor is determined by the unified perceptual experience or “gestalt” of a food that arises from the integration of sense of smell with several peripherally distinct sensory inputs, including taste, texture, viscosity, temperature, sight, and even the sound of foods or oral nociception (pain) [23, 24]. For instance, smell can arise from an external or internal source, this latter from inside the mouth during food consumption. Blankenship et al. demonstrated that internal odors share processing circuitry with taste and rapidly induce flavor preferences [25]. So, retro-nasal olfaction is probably the main determinant of flavor detection [26, 27]. Of particular interest is a recent study reporting that the 67% of COVID-19 patients experienced alterations in the retro-nasal smell; indeed, 42% reported a complete loss of retro-nasal smell perception, 35% reported that food had a different flavor than usual, and 17% reported a decreased retro-nasal smell perception. Moreover, 6% reported smell-specific perception, meaning some smell were perceived retro-nasally, while others were not [28].

Recently, our group has developed and validated a chemosensory test (namely, flavor test) to assess retro-nasal olfactory performance [29]. We have demonstrated that retro-nasal olfactory recognition in normal subjects is influenced by age and sex [30] and inversely correlates with Body Mass Index (BMI) [31]. Furthermore, patients with endocrine [29] and neurological diseases [32] had also a reduced flavor score.

Herein we investigated the effects of SARS-CoV-2 infection on chemosensory function, using both a self-reporting questionnaire on smell and flavor perception and a simplified flavor test that can be self-administered to isolated COVID-19 patients. Our data indicate that patients, after the infection, can recognize the tested aromas; however, they indicate to perceive flavors with a significant reduction in comparison to unaffected controls.

### Materials and methods

#### Studied population

All participants were adults (≥18 years of age) and gave their written informed consent to the study. The research was performed in keeping with Italian Bioethics Law and the Declaration of Helsinki. The flavor test has been approved by the Ethical Committee of the Federico II University of Napoli (IDs 253/13 and 93/19) and the study has been registered to ClinicalTrial.org (NCT04840966). Nineteen hospitalized and 37 home isolated COVID-19-positive patients were enrolled for the study. Diagnosis of COVID-19 was confirmed by PCR of nasopharyngeal swab. Only patients with no critical conditions and able to understand the protocol were recruited. No patient reported nasal obstruction nor previous nasal diseases. Fifty-five healthy COVID-19 swab-negative volunteers were enrolled as controls.

#### Questionnaires and self-evaluation

All the enrolled subjects received an instruction form with explanation of definition and differences among smell, taste, and flavor. Participants also received a self-evaluation questionnaire (fig. S1) to record personal data, anamnestic and current information on health status. Date of positivity for the nasopharyngeal swab for SARS-CoV-2 virus and list of symptoms (cold, sore throat, headache, muscle aches, fatigue, fever, gastrointestinal problems, dyspnea, cough, hyposmia, hypogeusia, other) were recorded for all the patients. Finally, patients were asked to score their subjective chemosensory function (smell and flavor) before and after COVID-19 using a 0–10 scale with 0 corresponding to “no smell/flavor perception” and 10 corresponding to “excellent smell/flavor perception.”

#### Modified flavor score test

The original flavor test, consisting in 20 aromas and one control solution [29], has been modified and simplified to be self-administered. Four aromas, among those mostly recognized in the healthy population [30], were selected. Each aroma was diluted in sucrose solutions according to the manufacturer’s instruction, as previously described [29]. Four tubes with 0.5ml of aromatic solutions (coffee, “pure olfactory”; peppermint, “mixed olfactory-trigeminal”; banana, “a complex chemical mixture” [33]) or water (control) were provided to the enrolled subjects. Aromas were kindly provided by the manufacturer GIOTTI (Enrico Giotti spa, Scandicci, Firenze, Italy).

All subjects received an instruction sheet where the test procedure was explained: participants were invited to transfer the content of each tube in their mouth, hold it for few seconds and afterward, recognize the aroma contained in the tube, and choose one of the five possible options. Participants were also asked to score on a 1 to 10 scale the intensity of perceived stimulus for each aroma, considering 0 corresponding to “no flavor perception” and 10 corresponding to “excellent flavor perception.”

#### Statistical analyses

Statistical analyses were performed using Prism 8 GraphPad software for macOS. Results were expressed as means ± standard deviation (SD) or median and interquartile ranges (IQR) for continuous variables. Mann-Whitney test was used for comparisons across groups. Frequencies were used for categorical variables, and comparisons were made using the Fisher’s exact test. Spearman’s correlation analysis was used to assess the occurrence of statistical associations between the variables under investigation. The level of significance was set at *α* = 0.05, with a two-sided level.

### Results

#### Sample characteristics

A total of 111 subjects (19 hospitalized [HOS] and 37 home-isolated [HI] COVID-19 patients, and 55 healthy controls [CTRL]) were enrolled in the study. Sample characteristics are shown in Table 1. The mean age for overall population was 41.52 ± 14.45 years. The HOS group presented a significantly higher age (*p* < 0.01) than HI and CTRL groups, while no age differences were found between HI and CTRL subjects. In the overall population median BMI was 25.82 kg/m^2^. No differences in BMI were present across the different study groups. The ratio between females and males enrolled in overall population, in CTRLS and HI, was similar. By contrast, in HOS group, there were more males (*n* = 17) than females (*n* = 2).

**Table 1:** General characteristic of studied population

	**Total (n = 111)**	**CTRL (n = 55)**	**HOS (n = 19)**	**HI (n = 37)**
Age (years)	41.52 ± 14.45	36.84 ± 12.01	54.21 ± 12.33^††,##^	41.97 ± 15.07^†^
BMI (kg/m^2^)	25.82 ± 4.10	25.79 ± 3.91	27.01 ± 3.74^†,#^	25.35 ± 4.51^†^
Sex (F/M)	52/59	33/22	2/17^††,##^	17/20^†^
Distance from swab (days)	6.05 ± 4.33	4.04 ± 2.22	8.11 ± 5.69^†,#^	7.97 ± 4.65^†^

Data are presented as mean ± SD for age, BMI, and distance from swab. *CTRL* healthy controls, *HI* home-isolated COVID-19, *HOS* hospitalized COVID-19

*p* vs CTRL: ^†^not significant, ^††^*p* < 0.01

*p* vs HI: ^#^not significant, ^##^*p* < 0.01

#### Questionnaires and self-assessed evaluation

The results of the questionnaire investigating COVID-19 symptoms are shown in Table 2. Several differences were present between the two groups of COVID-19 patients. In particular, HI presented more frequently than HOS cold, sore throat, headache, fatigue, and cough. The prevalence of STDs was reported to be similar among the two groups of patients.

**Table 2:** Number of subjects (and percentage) with specific symptoms in hospitalized and home-isolated COVID-19 patients

**Symptom**	**HOS**	**HI**	**Fisher’s exact test**
Cold	0 (0.00%)	9 (24.32%)	*p* < 0.05
Sore throat	0 (0.00%)	8 (21.62%)	*p* < 0.05
Headache	0 (0.00%)	17 (45.94%)	*p* < 0.01
Muscle aches	4 (21.05%)	14 (37.83%)	n.s.
Fatigue	2 (10.53%)	17 (45.94%)	*p* < 0.01
Fever	7 (36.84%)	24 (64.86%)	n.s.
Gastrointestinal problems	0 (0.00%)	5 (13.51%)	n.s.
Dyspnoea	1 (5.26%)	4 (10.81%)	n.s.
Cough	2 (10.53%)	16 (43.24%)	*p* < 0.05
Hyposmia	13 (68.42%)	24 (64.86%)	n.s.
Hypogeusia	14 (73.68%)	24 (64.86%)	n.s.
Other	1 (5.26%)	3 (8.11%)	n.s.

*HOS* hospitalized COVID-19, *HI* home-isolated COVID-19, *n.s.* not significant

**Fig. 1:**
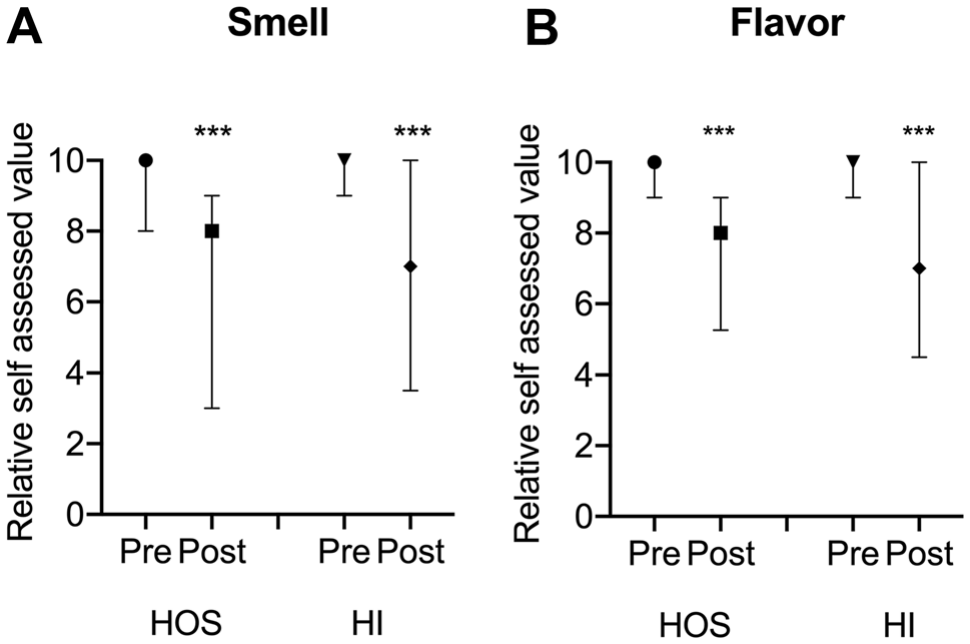
Quantitative self-estimated effect of SARS-CoV-2 infection on smell (**A**) and flavor (**B**). Chemosensory function was investigated asking the patients to score on a 1–10 scale their ability to recognize smell and flavors before (pre) and after (post) the infection. Median scores and IQR are shown. No differences across the HOS and HI groups were detected using the two-way ANOVA test. *HOS* hospitalized COVID-19, *HI* home-isolated COVID-19. ***p < 0.0001

The quantitative impact of COVID-19 on chemosensory function was investigated asking to the patients to score on a 1–10 VAS their ability to recognize smell and flavors before and after SARS-CoV-2 infection. Results are shown in Fig. 1. Positive COVID-19 patients indicated that smell perception changed from a median score of 9.0 (8.0–10.0) to 8.0 (3.0–9.0) in HOS (*p* < 0.0001), and from 9.0 (9.0–10.0) to 7.0 (3.5–10.0) in HI (*p* < 0.0001). The difference across the two groups was not different, as indicated by two-way ANOVA analysis (Fig. 1A). Similarly, after virus exposure, flavor perception changed from a median score of 10.0 (9.0–10.0) to 8.0 (5.25–9.0) (*p* < 0.0001) in HOS and from 10.0 (9.0–10.0) to 7.0 (4.5–10.0) (*p* < 0.0001) in HI patients, respectively (Fig. 1B). Again, no differences across the two groups were detected with the two-way ANOVA test.

To understand whether patients recovered from their symptoms, 3 months after the test they were interviewed by phone. STDs had a median last of 14 (9–27) days for smell and 14 (9–28) for flavor alterations, with no significant differences between the two groups. Interestingly, STDs after 3 months were still present in 33.33% (smell) and 11.76% (flavor) of HI patients, while a complete recovery was reported from all HOS patients.

#### Modified flavor test: recognition and intensity

The tested aromas (banana, coffee, and peppermint) were recognized in either HOS or HI COVID-19 patients, similarly to the CTRL group. Only water was slightly less recognized by HOS patients (*p* = 0.0497). The results are shown in Table 3.

**Table 3:** Percentage of correct recognition of the examined flavors in healthy controls, hospitalized, and home-isolated COVID-19 patients

	**Banana**	**Coffee**	**Peppermint**	**Water**
CTRL	78.18%	94.55%	94.55%	98.18%
HOS	94.74%	100%	94.74%	84.21%^‡^
HI	78.38%	81.08%	97.30%	94.59%

*CTRL* healthy controls, *HOS* hospitalized COVID-19, *HI* home-isolated COVID-19

^‡^*p* vs CTRL <0.05

**Fig. 2:**
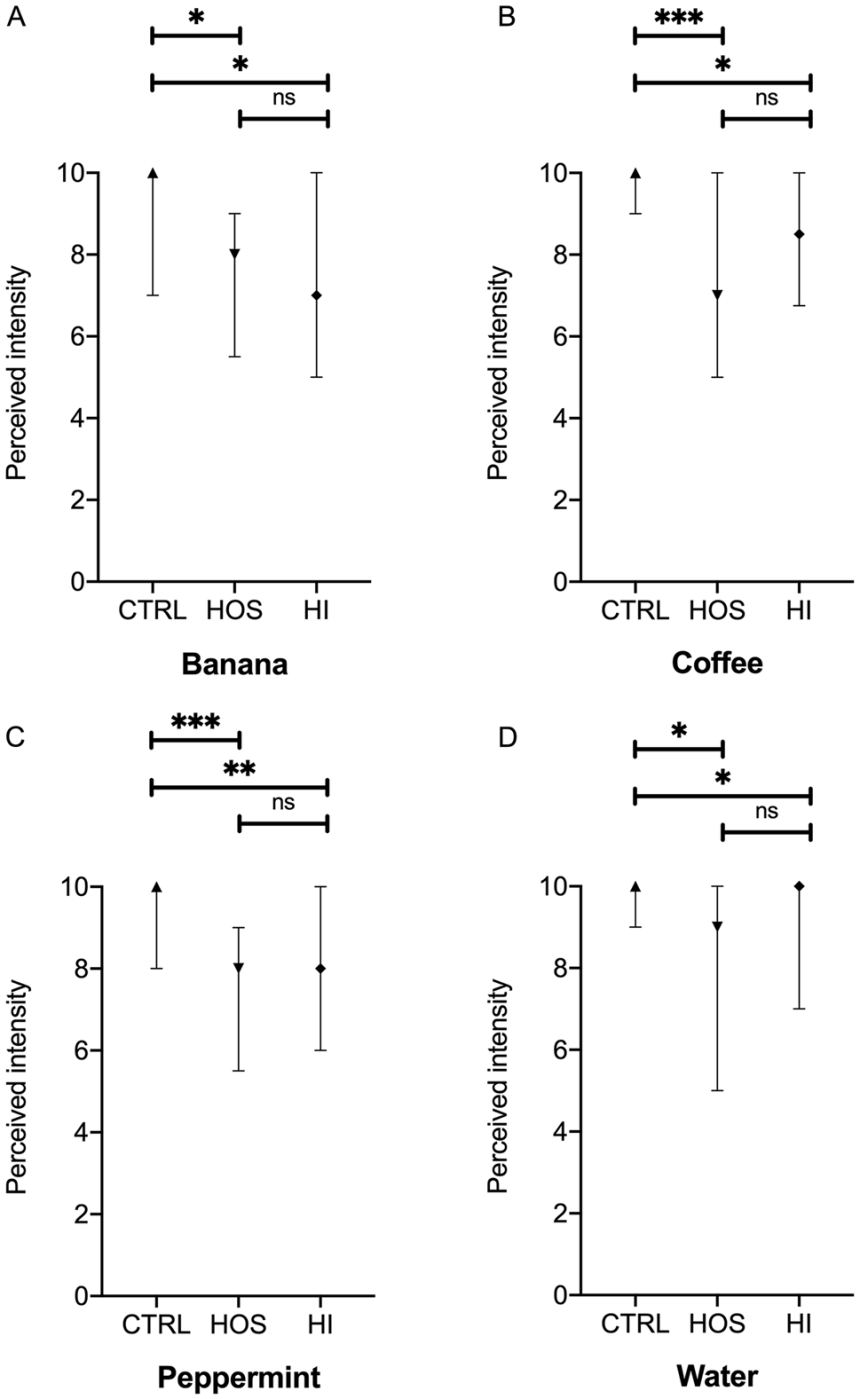
Perceived intensity of the tested aromas (**A** banana, **B** coffee, **C** peppermint, **D** water). Median scores and IQR are shown. *CTRL* healthy controls, *HOS* hospitalized COVID-19, *HI* home-isolated COVID-19 patients, *ns* not significant; *p < 0.05, **p < 0.01, ***p < 0.001

Finally, all participating subjects after recognizing each aroma were asked to score the intensity with which they perceived the flavor in the tubes using a 0–10 VAS scale. Results are shown in Fig. 2. Interestingly, although patients did not present qualitative alterations in terms of retro-nasal recognition, a significant quantitative impairment in chemosensory function was present in both HI and HOS COVID-19 patients compared to healthy controls, suggesting that SARS-CoV-2 virus infection determines a reduction in the intensity of flavor perception. The degree of retro-nasal olfactory intensity impairment was not significantly different between HI and HOS patients.

#### Correlations between self-evaluated STD and flavor test

**Fig. 3:**
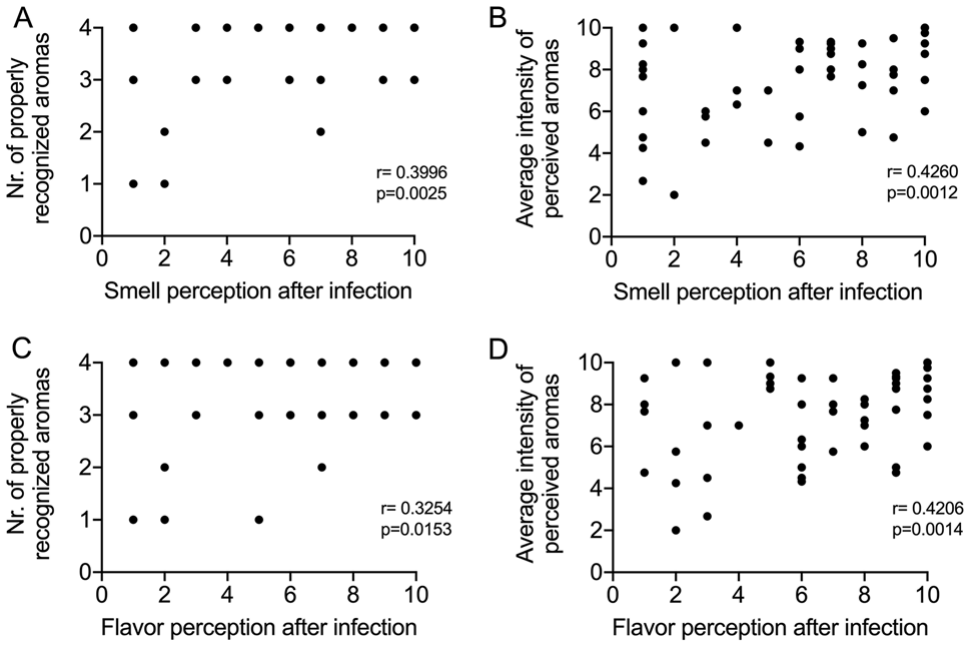
Correlation between self-estimated smell (**A** and **B**) and flavor (**C** and **D**) perception with number of properly recognized aromas (**A** and **C**) and average perceived intensity for the tested aromas (**B** and **D**). All correlations are significant. The analyses for each single aroma are shown in figs. S1 and  S2

To evaluate whether there are relationships between the perceived alterations of the chemosensory abilities determined by SARS-CoV-2 infection and the results of the modified flavor test, Spearman’s correlation analyses were performed. In details, statistical correlations were evaluated between the results of post-infection subjective chemosensory function (either smell and flavor) and the total score (0 to 4) determined by the sum of properly recognized flavors used in the test (Fig. 3A and C), the average of the intensity scores reported by the patients (Fig. 3B and D), and the intensity scores for each of the four tested aromas (figs. S2 and S3). Only correlation between intensity of the “water” aroma and post-infection self-evaluation of flavor was not significant (*p* = 0.052), while all the other correlations were significant.

Discussion and conclusions

Deterioration of chemical senses has been suggested as a relevant manifestation and one of the best predictors of SARS-CoV-2 infection [34–37]. Our study aimed to evaluate retro-nasal olfaction in hospitalized and home-isolated patients following SARS-CoV-2 infection using a self-assessment questionnaire and — for the first time — a self-administered flavor test. Overall, our results demonstrated a quantitative but not a qualitative impairment in flavor perception in COVID-19 patients, regardless the severity of the disease.

The original flavor test [29] has been simplified, with the great advantage to be self-administrable for COVID-19 patients in isolation, without any risk to health professionals. In this form of the test, a pure olfactory stimulus (coffee), a mixed olfactory-trigeminal stimulus (peppermint), and a complex chemical mixture (banana) [33, 38–40] were selected among the 20 aromas included in the original flavor test. In addition, these 3 stimuli were chosen mainly because easily recognizable in the large part of the general population during the validation test [30]. As reported in recent literature, the aroma categories were selected to contain both odors with little to no trigeminal sensation and odors with mixed sensations of odor and trigeminal in various degree [41, 42]. Water was included among the test solutions as control. Many of the chemosensory tests used in the recent literature are costly and time-consuming to administer, and often require in-person interactions of the health care provider with potentially infectious patients. The modified flavor test proposed in this study is cheap, quick, and self-administrable.

The results of the present study indicate that there are no qualitative differences in flavor recognition between COVID-19 patients (either HOS or HI) and unaffected controls. In contrast, the intensity of the perceived aromas was significantly lower in patients compared to controls, suggesting that SARS-CoV-2 infection is responsible for an impairment in chemosensory function. Chemosensory dysfunctions following COVID-19 seem to be different from other post-viral forms. A recent study has demonstrated that mechanisms of COVID-19 related olfactory dysfunction may reflect a specific involvement at the level of central nervous system [43]. Indeed, increasing evidence indicate that SARS-CoV-2 virus invades olfactory receptors and damages cell membrane of the first cranial nerve in nasal cavity and/or produces lesions in the central nervous system [44, 45]. Therefore, the direct effects of the virus on cortex, basal ganglia, and midbrain are associated with significant neuronal death [45]. This may account for reduce sensitivity to smells and flavors.

The lower intensity of perceived aromas was present regardless of the severity of the disease, since neither qualitative nor quantitative differences in flavor recognition were found between HOS and HI patients. This observation is discordant with previous reports, indicating that STDs are more frequently associated with mild forms of the disease [5, 22]. However, a phone interview performed 3 months after the test indicated that all HOS patients had a complete recovery from smell and flavor impairments, while these symptoms were still present in 33.33% and 11.75% of HI patients, respectively, suggesting that STDs last longer in patients with milder forms of the disease or, since the average age of HOS was than HI, in younger ones.

One of the critical points investigating chemosensory dysfunction is the difference that can be pointed out using self-evaluating or psychophysical tests. In healthy individuals the results of STDs evaluated with self-estimated methods are under-detected and under-reported in comparison to objective measures [46]. In contrast, their prevalence tends to be overestimated in COVID-19 patients [47]. Indeed, the self-evaluating assessments of STDs (with or without a Visual Analogic Scales [VAS]) [48, 49] can be influenced by several factors, including the bias determined by media attention to these symptoms.

The significant relationships observed between self-evaluated smell and flavor perception after SARS-CoV-2 infection and the results of the flavor test here proposed, indicate not only that our flavor test is cheap, quick to perform, and self-administrable, but also that the obtained results are superimposable to those obtained with a self-evaluating assessment of STDs. Most of the previous studies investigating STDs in COVID-19 patients were clinical surveys based on medical history and self-assessment. Only in some reports olfactory and gustatory functions following COVID-19 were measured with specific diagnostic tests [36, 50]. As previously indicated, this kind of tests may produce several *bias* and overestimate the relevance of alterations in chemosensory function. Indeed, as also recommended by recent papers, we suggested that a simple self-administered test could be a useful instrument to assess chemosensorial dysfunction in isolated/quarantined or hospitalized COVID-19 patients [13, 41]. Herein, for the first time, we propose a novel, simple, and reliable test that can be provided to COVID-19 patients for self-administration.

We are aware that this study has some limitations, first the absence of comparisons between retro-nasal and ortho-nasal olfaction as well as taste-testing. Nevertheless, it should be considered that evaluation of ortho-nasal olfaction requires the presence and the interaction with a healthy professional, with a relative long exposure to potentially infectious patients.

In addition, it should be noted that, in the examined study population, the HOS group was older than HI and CTRL groups, and that the prevalence of males was higher in HOS groups. These can be potential limitations, since age and sex may influence the flavor test results [30]. However, these data are in line with what reported in the literature. Indeed, severe clinical manifestations of the COVID-19 are more frequent in the elderly population, often requiring hospitalization [51, 52], and the prevalence of severe manifestations of COVID-19 is higher in males than in females [53].

In conclusion, in this study we propose a novel, simple, and self-administering chemosensory test to evaluate retro-nasal olfaction in isolated COVID-19-positive patients. The test demonstrated that the COVID-19 patients, regardless of the severity of the symptoms, can recognize aromatic solutions, but with a reduced intensity, compared to healthy controls. This suggests that the patients affected by SARS-CoV-2 virus present chemosensory impairment, and the modified “flavor test” could be a novel potential self-administering objective screening test STDs in COVID-19 patients.

## Supplementary Information

Below is the link to the electronic supplementary material.

Supplementary file1 (DOCX 290 KB)
