# Supplementary material for: Quantitative but not qualitative flavor recognition impairments in COVID-19 patients
Source: Ir J Med Sci. 2021 Sep 25;191(4):1759–66. doi: 10.1007/s11845-021-02786-x (PMC8475319; doi:10.1007/s11845-021-02786-x)
Supplement: Supplementary file 1 — Supplementary file1 (DOCX 290 KB) [file 11845_2021_2786_MOESM1_ESM.docx]

Supplementary Material

**Supplementary figure 1**

**Supplementary figure 1**. Questionnaire administered to patients to record clinical data

**Supplementary figure 2**

|  | **Banana** | **Coffee** | **Peppermint** | **Water** |
| --- | --- | --- | --- | --- |
| Spearman r | 0.4376 | 0.3338 | 0.5381 | 0.3516 |
| 95% confidence interval | 0.1566 to 0.6531 | 0.04627 to 0.5703 | 0.3033 to 0.7113 | 0.06632 to 0.5837 |
| P (two-tailed) | 0.0026 | 0.0204 | <0.0001 | 0.0143 |

**Supplementary figure 2**. Spearman’s correlation between self-estimated smell perception after SARS-CoV-2 infection and each of the tested aromas in the modified flavor test

**Supplementary figure 3**

|  | **Banana** | **Coffee** | **Peppermint** | **Water** |
| --- | --- | --- | --- | --- |
| Spearman r | 0.3997 | 0.4052 | 0.5191 | 0.2822 |
| 95% confidence interval | 0.1114 to 0.6259 | 0.1283 to 0.6234 | 0.2793 to 0.6980 | -0.01071 to 0.5306 |
| P (two-tailed) | 0.0065 | 0.0043 | <0.0001 | 0.0520 |

**Supplementary figure 3**. Spearman’s correlation between self-estimated flavor perception after SARS-CoV-2 infection and each of the tested aromas in the modified flavor test
